# Supplementary material for: Examination of the independent contribution of rheumatic heart disease and congestive cardiac failure to the development and outcome of melioidosis in Far North Queensland, tropical Australia
Source: PLoS Negl Trop Dis. 2022 Jul 18;16(7):e0010604. doi: 10.1371/journal.pntd.0010604 (PMC9292120; doi:10.1371/journal.pntd.0010604)
Supplement: S1 Table — (DOCX) [file pntd.0010604.s001.docx]

**S1 Table. Additional risk factors for melioidosis in patients with rheumatic heart disease and congestive cardiac failure**

3 rheumatic heart disease patients

- 1 had 2 additional risk factors (diabetes mellitus and hazardous alcohol consumption)
- 2 had 1 additional risk factor (1 had diabetes mellitus, 1 was receiving immunosuppressive therapy (prednisone and hydroxychloroquine))

26 congestive cardiac failure patients (none of whom had rheumatic heart disease)

- 2 had 4 additional risk factors (1 with diabetes mellitus, CKD, chronic lung disease and an active malignancy and 1 with hazardous alcohol use, immunosuppresion, chronic lung disease and an active malignancy)
- 1 had 3 additional risk factors (diabetes mellitus, hazardous alcohol use and chronic lung disease)
- 10 had 2 additional risk factors (4 had diabetes mellitus and CKD, 2 had chronic lung disease and hazardous alcohol use, 2 had diabetes mellitus and hazardous alcohol use, 1 had CKD and chronic lung disease, 1 had chronic lung disease and immunosuppression)
- 13 had 1 additional risk factor (5 had diabetes mellitus, 5 had chronic lung disease, 1 had hazardous alcohol use, 1 had active malignancy, and 1 had CKD)
