# Supplementary material for: Examination of the independent contribution of rheumatic heart disease and congestive cardiac failure to the development and outcome of melioidosis in Far North Queensland, tropical Australia
Source: PLoS Negl Trop Dis. 2022 Jul 18;16(7):e0010604. doi: 10.1371/journal.pntd.0010604 (PMC9292120; doi:10.1371/journal.pntd.0010604)
Supplement: S2 Table — (DOCX) [file pntd.0010604.s002.docx]

**S2 Table. Association between the presence of rheumatic heart disease and other predisposing factors**

|  | All n=392 ^a^ | RHD n=3 ^a^ | No RHD n=356 ^a^ | p ^b^ |
| --- | --- | --- | --- | --- |
| Diabetes | 201/377 (53.3%) | 2 (67.7%) | 186/356 (52.3%) | 1.0 |
| Hazardous alcohol use | 148/360 41.1%) | 1 (33.3%) | 143/350 (40.9%) | 1.0 |
| Chronic lung disease | 69/363 (19.0%) | 0 | 68/355 (19.2%) | 1.0 |
| Chronic kidney disease | 55/375 (14.5%) | 0 | 51/355 (14.4%) | 1.0 |
| Malignancy | 35/362 (9.7%) | 0 | 35/355 (9.9%) | 1.0 |
| Immunosuppression | 52/243 (21.4%) | 1 (33.3%) | 51/242 (21.1%) | 1.0 |

In only 359 patients could the presence - or absence - of rheumatic heart disease be confidently determined

^a^ The denominator for each of the risk factors varies due to incomplete data for some of the 197 patients presenting before October 2016 in whom data were collected retrospectively.

^b^ Due to the very small number of rheumatic heart disease patients, Fisher’s exact test was used for this comparison.
