# Supplementary material for: Examination of the independent contribution of rheumatic heart disease and congestive cardiac failure to the development and outcome of melioidosis in Far North Queensland, tropical Australia
Source: PLoS Negl Trop Dis. 2022 Jul 18;16(7):e0010604. doi: 10.1371/journal.pntd.0010604 (PMC9292120; doi:10.1371/journal.pntd.0010604)
Supplement: S3 Table — (DOCX) [file pntd.0010604.s003.docx]

**S3 Table. Association between the presence of congestive cardiac failure and other predisposing factors**

|  | All n=392 ^a^ | CCF n=26 ^a^ | No CCF n=327 ^a^ | p |
| --- | --- | --- | --- | --- |
| Diabetes | 201/377 (53.3%) | 13/26 (50.0%) | 168/324 (51.9%) | 0.86 |
| Hazardous alcohol use | 148/360 41.1%) | 6/26 (23.1%) | 134/317 (42.3%) | 0.06 |
| Chronic lung disease | 69/363 (19.0%) | 12/25 (48.0%) | 55/321 (17.1%) | <0.001 |
| Chronic kidney disease | 55/375 (14.5%) | 8/26 (30.8%) | 42/324 (13.0%) | 0.02 |
| Malignancy | 35/362 (9.7%) | 3/25 (12.0%) | 32/321 (10.0%) | 0.73 |
| Immunosuppression | 52/243 (21.4%) | 2/20 (10.0%) | 48/221 (21.7%) | 0.27 |

In only 353 patients could the presence of congestive cardiac failure be confidently determined.

^a^ The denominator for each of the risk factors varies due to incomplete data for some of the 197 patients presenting before October 2016 in whom data were collected retrospectively.
