# Supplementary material for: Examination of the independent contribution of rheumatic heart disease and congestive cardiac failure to the development and outcome of melioidosis in Far North Queensland, tropical Australia
Source: PLoS Negl Trop Dis. 2022 Jul 18;16(7):e0010604. doi: 10.1371/journal.pntd.0010604 (PMC9292120; doi:10.1371/journal.pntd.0010604)
Supplement: Attachment — Submitted filename: PNTD-D-22-00405 response to reviewer.docx [file pntd.0010604.s004.docx]

23 June 2022

Dear Prof Vinetz,

Thank you for the opportunity to submit a revised version of our manuscript. We would like to thank you and the reviewer, Professor Dance, for the time that you have taken to review our manuscript and your suggestions for its improvement. Please find below our point-by-point responses to your comments. The document is colour-coded (reviewer comments in blue, our responses in black) to ease legibility.

Reviewer's Responses to Questions

Reviewer #1: David Dance

Key Review Criteria Required for Acceptance?
As you describe the new analyses required for acceptance, please consider the following:

Methods
-Are the objectives of the study clearly articulated with a clear testable hypothesis stated?
-Is the study design appropriate to address the stated objectives?
-Is the population clearly described and appropriate for the hypothesis being tested?
-Is the sample size sufficient to ensure adequate power to address the hypothesis being tested?
-Were correct statistical analysis used to support conclusions?
-Are there concerns about ethical or regulatory requirements being met?

Reviewer #1: No new analyses required

**Response:** We are happy to hear that Professor Dance is happy with the presentation of our methods.

Results
-Does the analysis presented match the analysis plan?
-Are the results clearly and completely presented?
-Are the figures (Tables, Images) of sufficient quality for clarity?

Reviewer #1: Results clearly presented

**Response:** We are happy to hear that Professor Dance is happy with the presentation of our results.

Conclusions
-Are the conclusions supported by the data presented?
-Are the limitations of analysis clearly described?
-Do the authors discuss how these data can be helpful to advance our understanding of the topic under study?
-Is public health relevance addressed?

Reviewer #1: Conclusions and limitations clearly described

**Response:** We are happy to hear that Professor Dance is happy with the presentation of our conclusions.
Editorial and Data Presentation Modifications?
Use  this section for editorial suggestions as well as relatively minor modifications of existing data that would enhance clarity. If the only modifications needed are minor and/or editorial, you may wish to recommend “Minor Revision” or “Accept”.

Reviewer #1: A few very minor edits need - for example commas not necessary in lines 135, 144, 155 and 356; insert gap before parentheses in first column of 'Hazardous alcohol use' in Table 2.

**Response:** We have deleted the commas in lines 135, 144, and 356 and we have inserted a gap before the parentheses in first column of 'Hazardous alcohol use' in Table 2 as suggested. We could not find a comma in line 155.

Summary and General Comments
Use  this section to provide overall comments, discuss strengths/weaknesses of the study, novelty, significance, general execution and scholarship. You may also include additional comments for the author, including concerns about dual publication, research ethics, or publication ethics. If requesting major revision, please articulate the new experiments that are needed.

Reviewer #1: This well written paper looks at whether there was a significant association between melioidosis and rheumatic heart disease and congestive cardiac failure in Far North Queensland.  The reasons why such as association was not seen in this cohort as opposed to that in Darwin is discussed.

**Response:** We are happy to hear that Professor Dance, a leader in the global melioidosis academic community, appears happy with our manuscript and has relatively few suggestions for its enhancement.
